# Iroquois homeobox 2 suppresses cellular motility and chemokine expression in breast cancer cells

**DOI:** 10.1186/s12885-015-1907-4

**Published:** 2015-11-11

**Authors:** Stefan Werner, Hauke Stamm, Mutiha Pandjaitan, Dirk Kemming, Benedikt Brors, Klaus Pantel, Harriet Wikman

**Affiliations:** Department of Tumor Biology, University Medical Center Hamburg-Eppendorf, Martinistrasse 52, 20246 Hamburg, Germany; European Laboratory Association, Ibbenbüren, Germany; Department of Applied Bioinformatics (G200), German Cancer Research Center (DKFZ), Heidelberg, Germany; National Center for Tumor Diseases (NCT), 69120 Heidelberg, Germany; German Consortium for Translational Cancer Research (DKTK), 69120 Heidelberg, Germany

**Keywords:** Breast cancer, Metastasis, Migration, IRX2, Chemokines, Disseminated tumor cells

## Abstract

**Background:**

Disseminated tumor cells (DTCs) can be detected using ultrasensitive immunocytochemical assays and their presence in the bone marrow can predict the subsequent occurrence of overt metastasis formation and metastatic relapse. Using expression profiling on early stage primary breast tumors, low IRX2 expression was previously shown to be associated with the presence of DTCs in the bone marrow, suggesting a possible role of IRX2 in the early steps of metastasis formation. The purpose of this study is to gain insights into the significance of IRX2 protein function in the progression of breast cancer.

**Methods:**

To assess the physiological relevance of IRX2 in breast cancer, we evaluated IRX2 expression in a large breast cancer cohort (*n* = 1992). Additionally, constitutive IRX2 over expression was established in BT-549 and Hs578T breast cancer cell lines. Subsequently we analyzed whether IRX2 overexpression effects chemokine secretion and cellular motility of these cells.

**Results:**

Low IRX2 mRNA expression was found to correlate with high tumor grade, positive lymph node status, negative hormone receptor status, and basal type of primary breast tumors. Also in cell lines low IRX2 expression was associated with mainly basal breast cancer cell lines. The functional studies show that overexpression of the IRX2 transcription factor in basal cell lines suppressed secretion of the pro-metastatic chemokines and inhibited cellular motility but did not influence cell proliferation.

**Conclusion:**

Our results imply that the IRX2 transcription factor might represent a novel metastasis associated protein that acts as a negative regulator of cellular motility and as a repressor of chemokine expression. Loss of IRX2 expression could therefore contribute to early hematogenous dissemination of breast cancer by sustaining chemokine secretion and enabling mobilization of tumor cells.

**Electronic supplementary material:**

The online version of this article (doi:10.1186/s12885-015-1907-4) contains supplementary material, which is available to authorized users.

## Background

Metastasis - the main cause of cancer related deaths - is a complex multi-step process [1]. A key event is the systemic spread of single tumor cells from the primary lesion through the blood circulation into distant organs. The presence of disseminated tumor cells (DTC) in the bone marrow (BM) of cancer patients is an independent predictor of metastatic relapse in breast cancer and other solid tumors [2, 3]. Despite progress in the classification of tumors and the identification and characterization of disseminated tumor cells in BM of cancer patients [4], the early events of the metastatic cascade, which leads to shedding of single tumor cells from the primary tumor mass still remains elusive.

The transcription factor Iroquois Homeobox 2 (IRX2) is a member of the Iroquois homeobox gene family. Members of this family appear to play multiple roles during pattern formation of vertebrate development [5–7]. In the breast tissue, IRX2 is expressed in ductal and lobular epithelium, but not in myoepithelium and it is suggested to function in linage-specific epithelial cell differentiation [8]. The relevance of the IRX2 transcription factor throughout cancer progression is somewhat contradictory. Amplification of the *IRX2* chromosomal locus on 5p15.33 has been identified in breast and soft tissue sarcomas [9, 10]. In breast cancer this amplification may coexists with an activating mutation of the *PIK3CA* gene [9]. In osteosarcomas knockdown of IRX2 inhibits cell proliferation and invasion [11], and elevated IRX2 expression is correlated with worse outcome and age in infant acute lymphoblastic leukemia [12]. Thus, these studies suggest a possible oncogenic function for the IRX2 protein, especially in malignant cells of mesenchymal origin. In contrast, other studies have shown that hypermethylation of *IRX2* promoter region frequently occurs in lung squamous cell and adenocarcinomas [13, 14]. Also one study showed that CpG islands in the *IRX2* gene were significantly more methylated in luminal A in comparison to basal tumors [15]. Most of these studies have not performed functional validation of the exact biological role of the IRX2 in tumor progression. We have recently shown that low *IRX2* expression is associated with the presence of DTCs in the bone marrow of breast cancer patients [16], suggesting a possible role of IRX2 as a metastasis suppressor protein in breast cancer.

Although many of the early events of tumor cell dissemination and metastasis formation remain unclear, different studies emphasize the importance of chemokines in the microenvironment of the primary tumor and the site of metastasis for cancer cell dissemination and metastatic outgrowth [17]. For instance the expression of the chemokine CCL5 (RANTES) can be correlated with progressive disease in breast cancer [18] and bone metastasis of breast cancer cells is depending on signaling through the associated receptor CCR5 [19]. Coincidently CCR5 antagonists block metastasis formation of the breast cancer cell line MDA-MB-231 in mice, providing evidence for a key role of CCL5/CCR5 in the invasiveness of basal breast cancer cells [20]. Although accumulating evidence emphasizes the central impact of chemokines on metastasis formation in breast cancer [21, 22], the mechanism for elevated levels of tumor cell derived chemokines secretion remains poorly understood.

In this study, we aimed to validate the clinical importance of IRX2 expression and to gain insights into the significance of IRX2 expression in the progression of breast cancer. The obtained data provide further evidence for IRX2 as a potential metastasis suppressor as ectopic IRX2 expression diminished secretion of different chemokines and acts as negative regulator of cellular motility of breast cancer cells.

## Results

### Expression of IRX2 in primary breast tumors

We previously found that low *IRX2* gene expression in primary breast tumors is associated with the presence of DTCs in the bone marrow [16]. Low *IRX2* was also associated with shortened survival of breast cancer patients in one analyzed breast cancer data set [16]. To further investigate the patho-physiological relevance of *IRX2* gene expression in breast cancer, we evaluated IRX2 gene expression in a large publically available patient cohort comprised of 1992 patients (Table 1). We found that *IRX2* is associated with several clinical prognostic factors. Low *IRX2* mRNA expression was found to be correlated with high tumor stage (*p* = 0.004), high tumor grade (*p* < 0.001) and the presence of lymph node metastasis (*p* = 0.044). On the other hand, low *IRX2* mRNA expression was found to be significantly correlated with low expression of both the estrogen (*p* = 0.001) and the progesterone (*p* < 0.001) steroid receptors. Surprisingly, lower *IRX2* mRNA expression was also significantly correlated with smaller tumor size (*p* = 0.05). Finally we found that high IRX2 expression is associated with the luminal A molecular subtype and that low *IRX2* expression is significantly more frequent in tumors classified as basal and luminal B (*p* < 0.001). Taken together these analyses clearly show that low *IRX2* expression is correlated with different parameters of poor prognosis, indicating that loss of *IRX2* expression is associated with less differentiated and more aggressive breast tumors. Nonetheless, no significant correlation between low *IRX2* expression and shortened survival was found in this data set (data not shown).

**Table 1:** Analysis of IRX2 mRNA expression in primary breast tumors. IRX2 expression was determined in one large published expression data set [29] and correlated to the indicated clinico-pathological parameters

		IRX2 low		IRX2 high		*P*-value
		n	%	n	%	
All patients		998	50.1	994	49.9 %	
Histology						<0.001
	Ductal	809	82.7	744	76.5	
	Lobular	47	4.8	101	10.4	
	Ductolobular	42	4.3	48	4.9	
	Others	69	7.1	76	7.8	
Tumor stage						0.004
	1	174	31.8	198	39.4	
	2	308	56.2	271	53.9	
	3 + 4	66	12.1	34	6.8	
Grade						<0.001
	1	59	6.2	111	11.7	
	2	352	36.9	423	44.7	
	3	544	57.0	413	43.6	
Lymph node status						0.044
	N0	503	50.4	546	54.9	
	N+	495	49.6	448	45.1	
Tumor size						
	<2 cm	407	43.3	499	45.8	0.050
	>2 cm	578	58.7	532	54.2	
ER expression						0.001
	Negative	268	26.9	206	20.7	
	Positive	730	73.1	788	79.3	
PR expression						<0.001
	Negative	540	54.1	403	40.5	
	Positive	458	45.9	591	59.5	
HER2 expression						0.735
	Negative	876	87.8	867	87.2	
	Positive	122	12.2	127	12.8	
Subtype						<0.001
	Basal	206	20.7	125	12.6	
	HER2	121	12.2	119	12.0	
	LumA	303	30.5	418	42.2	
	LumB	292	29.3	200	20.2	
P53 mutation status						0.454
	Mutant	56	12.9	43	11.1	
	wt	378	87.1	343	88.9	

### Expression of IRX2 in breast cancer cell lines

**Fig. 1:**
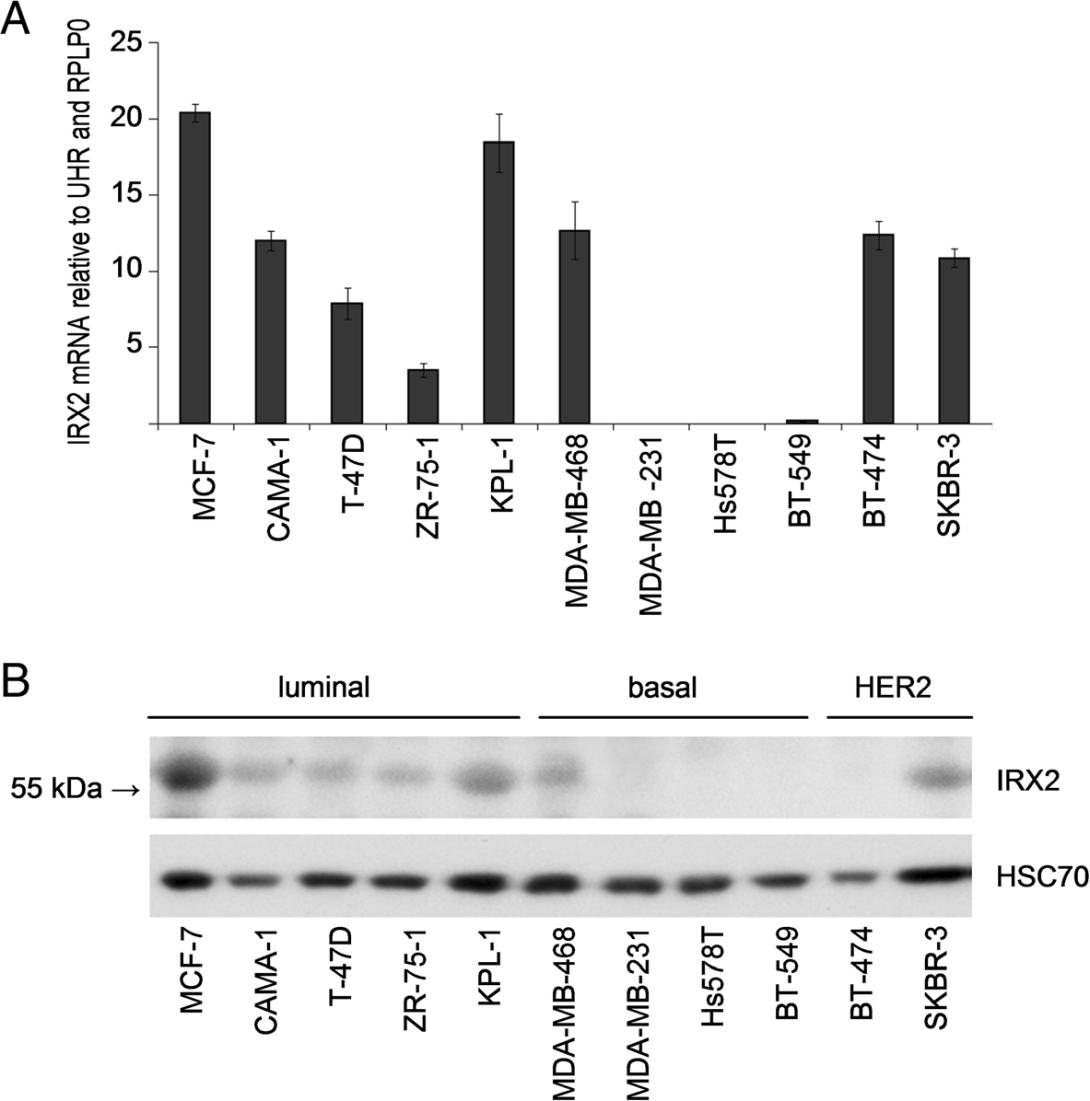
Analysis of IRX2 mRNA and protein expression in breast cancer cell lines. **a** Quantitative gene expression analysis of *IRX2* in different breast cancer cell lines. The data shown are the average fold change normalized to RPLP0 and UHR expression of three independent experiments; the error bars represent the standard deviation of the mean. **b** IRX2 protein expression in the same panel of human breast carcinoma cell lines was determined by Western blot analysis using a polyclonal RAI2-specific antibody that recognizes an internal IRX2 epitope. Equal loading was demonstrated using an antibody recognizing HSC70

To further investigate the significance of IRX2 expression in breast cancer, we evaluated IRX2 expression in a panel of breast cancer cell lines. *IRX2* mRNA expression was detected in eight out of 11 breast cancer cell lines at variable levels, as determined by quantitative real-time PCR analysis (Fig. 1a). The IRX2 protein expression was determined by Western blot analysis (Fig. 1b) in the same set of cell lines using a custom-made IRX2-specific polyclonal antibody. The level of IRX2 protein expression corresponds well with the level of *IRX2* mRNA detected in these cells. In line with a potential metastasis suppressing function of IRX2, the poorly differentiated, highly metastatic basal breast cancer cell lines MDA-MB-231, Hs578T and BT-549 were completely negative for IRX2 protein expression. IRX2 expression was found in the luminal A cell lines (MCF7, CAMA-1, T-47D, ZR-75-1 and KPL1) as well as in the HER2 over expressing cell lines SKBR-3 and BT474. In BT-474 we found IRX2 transcript but no protein expression.

### Analysis of IRX2 regulated network

Existing functional studies have so far described the biological function of IRX2 mainly in the context of pattern formation during embryonic development [5, 6]. To obtain evidence about the biological function of the IRX2 transcription factor in breast epithelial cells and to identify genes that might represent direct transcriptional targets, we used SAM-analysis (Significance Analysis of Microarrays) on a large published data set of breast cancer patients (GSE6532). Altogether 17 transcripts were found significantly inversely correlated with IRX2 expression and 43 transcripts were concurrently upregulated in patients with high IRX2 expression (Additional file 1: Table S1). Gene ontology analysis [23] was carried out on these 60 most correlated and inversely correlated genes Interestingly, 6 out of 60 defined genes belonged to “chemokine signaling pathway” (*p*-value: <0.001; Benjamini: 0.001). Among the inversely correlated genes were four chemokines, *CCL5*, *CXCL9*, *CXCL10* and *CXCL11* indicating that IRX2 might be actively involved in the regulation of chemokine secretion by mediating the transcriptional repression of these chemokines in breast cancer cells.

### IRX2 protein expression represses chemokine secretion of breast cancer cells

**Fig. 2:**
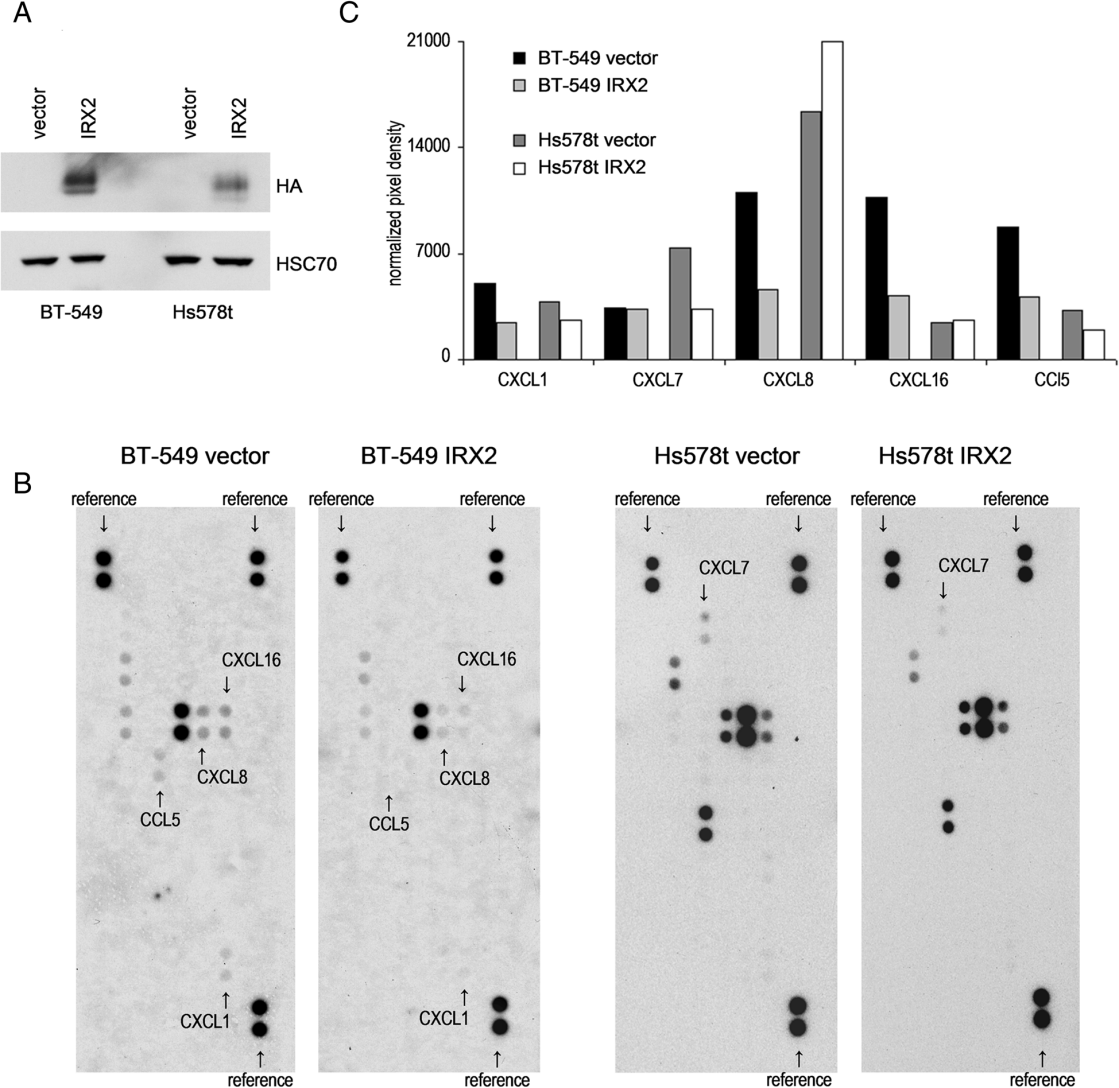
Analysis of IRX2 expression and chemokine secretion in breast cancer cell lines over expressing IRX2. **a** Recombinant IRX2 protein expression in BT-549 and Hs578T cells that were transduced with retro-virus containing HA-tagged IRX2-cDNA. IRX2 protein expression was determined by Western blot analysis using a HA-specific antibody. Equal loading was demonstrated using an antibody recognizing HSC70. **b** Results from Proteome Profiler Antibody Array for determination of relative chemokine secretion. Cell culture supernatants from BT-549 and Hs578t cells over expressing the IRX2 protein were analyzed and compared with the vector control supernatants. **c** Normalized pixel intensities from Proteome Profiler Antibody Arrays. Individual signal intensities were measured using ImageJ software and normalized to the mean signal intensities of all reference spots

To experimentally validate the possible role of IRX2 as a metastasis suppressing protein and repressor of migration and chemokine expression, we stably expressed a c-terminal HA-tagged IRX2 protein in IRX2-deficient BT-549 and Hs578t breast cancer cell lines. Successful ectopic IRX2 protein expression in both cell lines was validated by Western Blot analysis using a HA-specific antibody (Fig. 2a).

To investigate whether ectopic IRX2 expression has an influence on chemokine secretion, we analyzed conditioned cell culture media from BT-549 and Hs578t cells with a chemokine antibody array. In cell culture supernatants taken from BT-549 cells we found differences in the presence of CCL5, CXCL1, CXCL8 and CXCL11. Remarkably, the expression of all four chemokines was considerably lower in BT-549 cells overexpressing the IRX2 protein confirming the potential role of IRX2 in the control of chemokine expression (Fig. 2b/c). In cell culture supernatants taken from Hs578t vector control cells we found more CXCL7 expression in comparison to supernatants taken form cells overexpressing IRX2 (Fig. 2b/c). Interestingly, we also found reduced expression of CCL5 in Hs578t cells overexpressing IRX2, tough the overall expression of CCL5 was low abundant in Hs578t cells.

### Migration inhibitory activities of IRX2 in human breast cancer cells

**Fig. 3:**
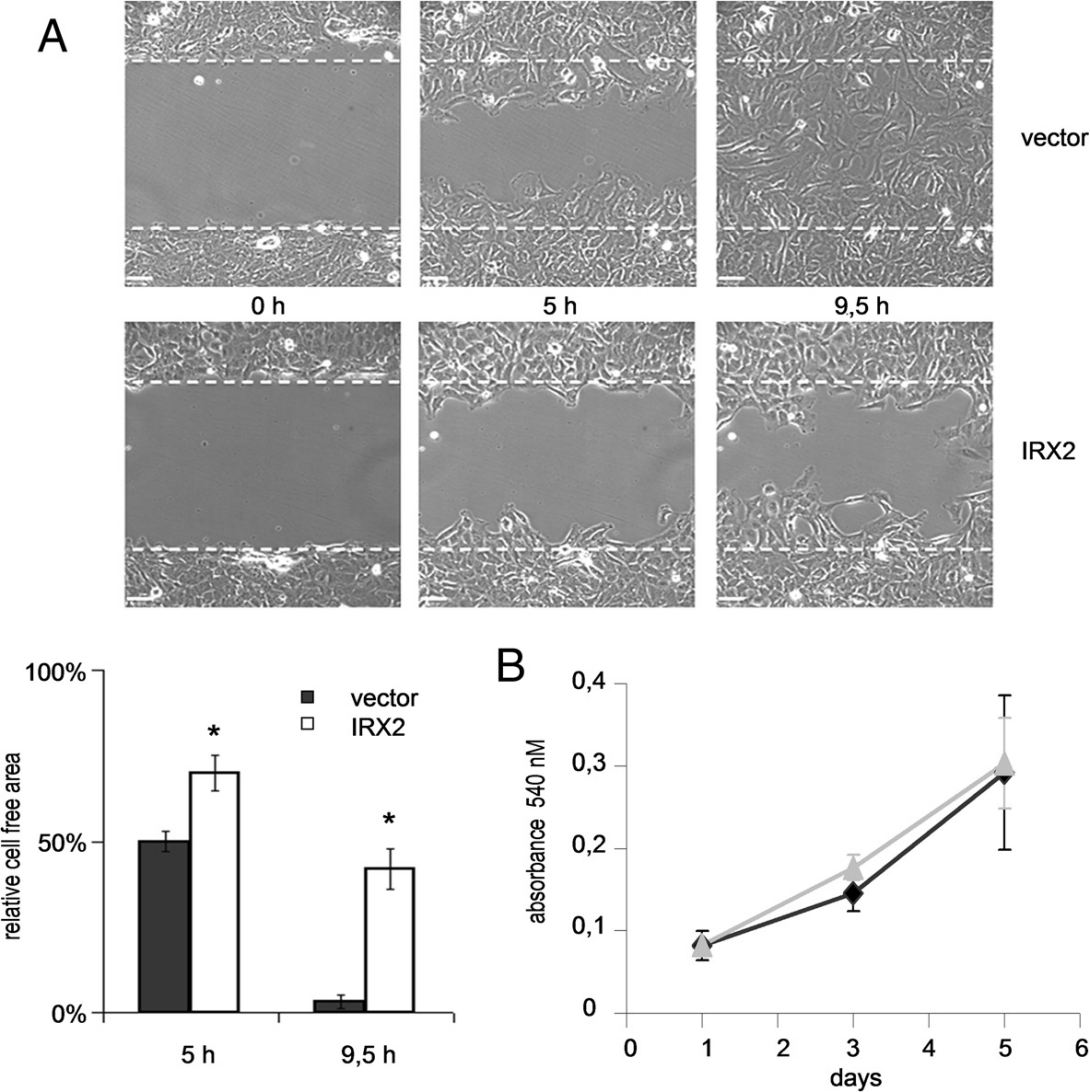
Analysis of cell migration and proliferation. **a** Migration analysis of BT-549 cells over expressing IRX2 and from control cells by wound healing assay. Closure of wounded cell layer was examined by time-lapse videomicroscopy and evaluated using the Volocity 6 software. **b** Analysis of cell proliferation of BT-549 cells over expressing IRX2 and control cell lines by MTT assay. Five thousand cells were plated in quadruplicates and incubated under normal culture conditions for the indicated time span before measurement. Error bars represent the standard deviation of the mean of three independent experiments

To further explore the possible role for IRX2 in tumor progression, we investigated the ability of IRX2 expression to modify cell migration and proliferation of breast cancer cells. Migration of BT-549 cells was determined by performing a wound healing (scratch) assay (Fig. 3a). At 5 h, in the presence of 10 % FCS, vector-transduced BT549 cells closed half of the wound, whereas BT-549 cells with ectopic IRX2 protein expression only closed 30 %. After 9.5 h vector transduced BT-549 cells closed the entire wound whereas the IRX2 expressing cells closed 58 % (*P* < 0.001). These results clearly show that ectopic expression of IRX2 protein inhibits the migration of BT-549 cells. The difference was not caused by a different proliferation rate as shown in Fig. 3b. We could not detect any effect on cell proliferation in cells over expressing the IRX2 protein.

### Validation of chemokine transcript expression in cells over expressing IRX2

**Fig. 4:**
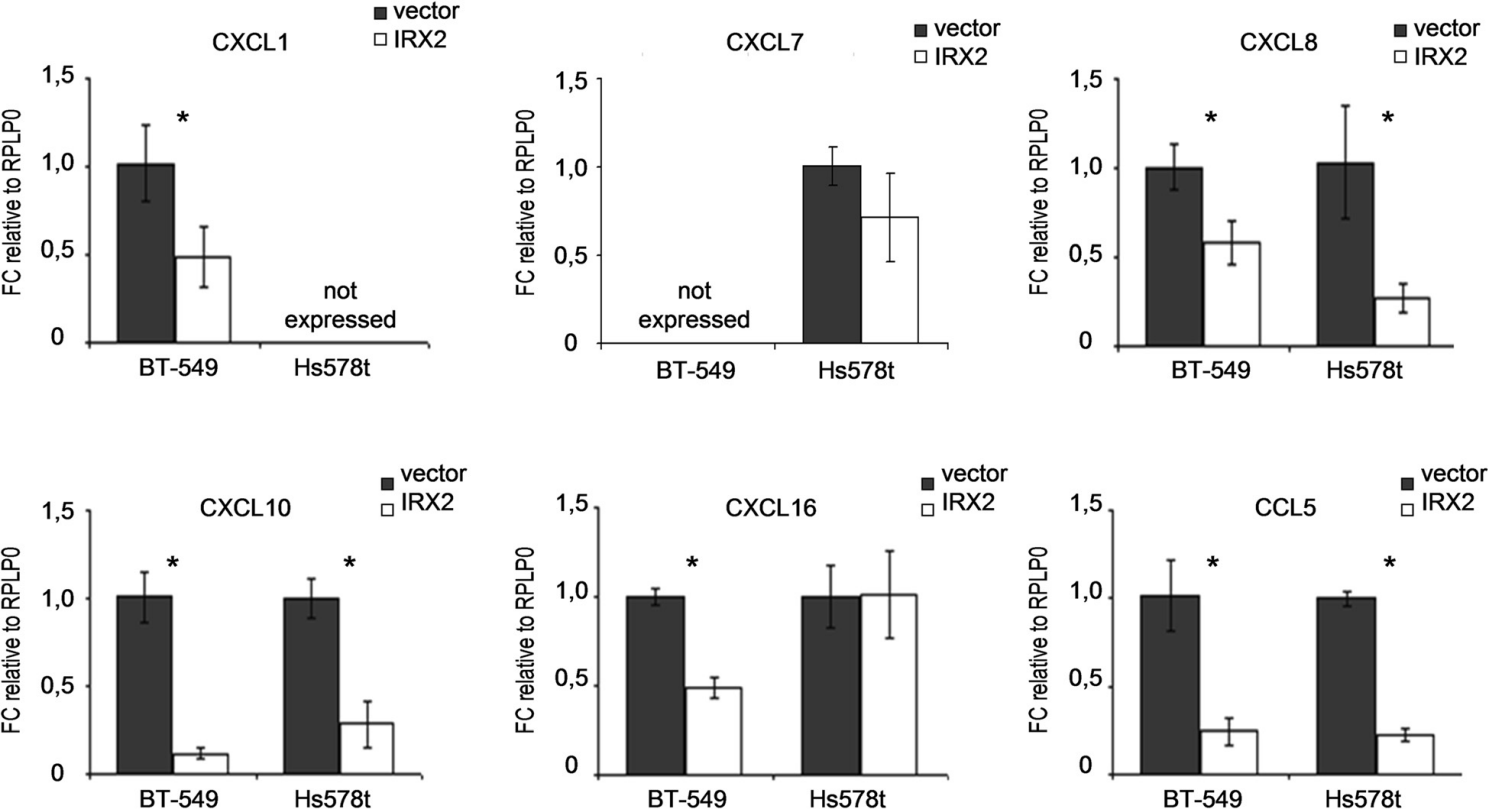
Validation of mRNA expression of different chemokines in BT-549 and Hs578T cells over expressing IRX2. Quantitative gene expression analysis of the indicated chemokines. The data shown are the average fold change normalized to RPLP0 and parental cell line expression of three independent experiments; the error bars represent the standard deviation of the mean. *P*-values are calculated with the two-sided Student’s *t*-test (*, *p* < 0.05)

To validate whether the IRX2 protein represses chemokine expression in BT549 and Hs578T cells, we examined mRNA expression of the potential IRX2 targets *CCL5, CXCL1, CXCL7, CXCL8, CXCL9, CXCL10, CXCL11* and *CXCL16* by qPCR analysis (Fig. 4). *CCL5, CXCL8* and *CXCL10* were found to be expressed in both cell lines at abundant levels and, furthermore, we found a concordant downregulation of all three chemokines in the two cell lines that over express IRX2. *CXCL16* was also found to be expressed in both cell lines but IRX2 overexpression led only to a reduced *CXCL16* expression in BT-549 cells (Fig. 4). *CXCL1* was found not to be expressed in Hs578T cells, whereas *CXCL1* mRNA expression could be detected in BT-549 cells and its expression is reduced in BT-549 cells over expressing IRX2. *CXCL7* was found not to be expressed in BT-549 cells, whereas *CXCL7* mRNA expression was detected in Hs578t cells. However, the qPCR analysis showed no significant reduced *CXCL7* expression in Hs578t cells over expressing IRX2. *CXCL9* and *CXCL11* were not found to be expressed in the two analyzed cell lines. We also investigate whether the IRX2 transcription factor has a direct effect on CCL5 promoter activity and conducted a reporter gene assay using the proximal human CCL5 promoter fragment linked to the firefly luciferase gene. Luciferase activity was also shown to be significantly lower in presence of the IRX2 protein in BT-549 cells (Additional file 2: Figure S1).

### Validation of migration inhibitory activities of IRX2

**Fig. 5:**
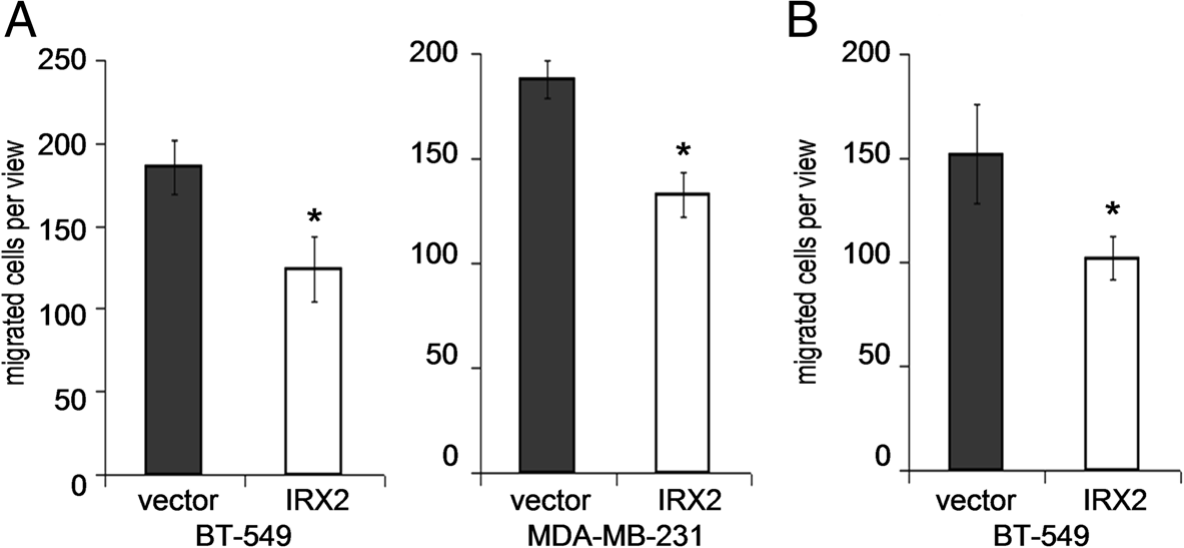
Analysis of cell migration. **a** Migration analysis of parental BT-549 and MDA-MB-231 cells using Boyden chamber assay. Cell culture supernatants from BT-549 cells over expressing IRX2 or from control cell lines were used as chemoattractant for 24 h. Error bars represent the standard deviation of the mean of three independent experiments done in triplicates. *P*-values were calculated by the two-sided Student’s *t*-test (*, *p* < 0.05). **b** Migration analysis of BT-549 cells over expressing IRX2 and from control cells using Boyden chamber assay Standard cell culture medium was used as chemoattractant for 24 h. Error bars represent the standard deviation of the mean of three independent experiments done in triplicates. *P*-values were calculated by the two-sided Student’s *t*-test (*, *p* < 0.05)

As IRX2 suppresses the secretion of different chemokines, we used the Boyden chamber assay to analyze the effect of conditioned media obtained from either vector- or IRX2-transduced BT-549 cells as a potential chemoattractant for parental BT-549 or MDA-MB-231 cells, respectively. Both cell lines were seeded in starvation medium into the upper chambers. The bottom wells held conditioned medium from either vector- or IRX2-transduced cells. After 24 h the number of migrated cells was determined. Cell counts of migrated cells of both cell lines were decreased when conditioned medium from IRX2 expressing cells was used as chemoattractant. These results demonstrate that IRX2 over expression has a negative effect on autocrine stimulation of cellular motility (Fig. 5a). We further analyzed the potential inhibitory effects of IRX2 over expression on migration in a Boyden chamber assay. We seeded either vector- or IRX2-transduced BT-549 cells into the upper chambers and in the lower chamber DMEM containing 10 % FCS was used as a chemoattractant. Also in this experiment, the number migrated cells were significantly decreased for cells expressing the IRX2 transcription factor in comparison to vector control cells (Fig. 5b).

## Discussion

In this study we sought to examine the significance of IRX2 expression in the progression of breast cancer. In our previous study we showed that a low *IRX2* gene expression in early stage primary breast tumors is associated with the presence of DTCs in the bone marrow and was also associated with shortened survival of breast cancer patients in one analyzed breast cancer data set [16], indicating a possible function of the IRX2 protein as a metastasis suppressing protein. In contrast, it has been reported that some breast cancers exhibit high levels of IRX2 expression [9] and that an amplification of the *IRX2* genomic locus frequently coexist with an activating mutation of the *PIK3CA* gene in breast cancer [9], pointing also to a possible oncogenic function of the IRX2 protein in cell proliferation. Furthermore, immunohistochemical staining of 85 breast tumors showed expression of the IRX2 protein in cancerous breast tissue and revealed a significant correlation of IRX2 protein expression only with tumor size, suggesting a possible oncogenic function for the IRX2 protein in breast cancer [9]. On the other hand, we found that high *IRX2* mRNA expression correlates with different parameters that define well differentiated breast tumors and good clinical outcome, like positive hormone receptor status as well as low tumor stage and grade. Also high IRX2 expression was found to be inversely correlated with lymph node status, a known indicator of an unfavorable prognosis. Taken together our results suggest that low *IRX2* mRNA expression is a characteristic of tumors that have acquired an aggressive phenotype. Thus, IRX2 could exhibit dual functions in the progression of breast cancer, i.e., in the early stages of tumor development elevated IRX2 expression might contribute to tumor cell proliferation and transformation. This might also depend on concomitant genetic lesions like the described activating mutation of the *PIK3CA* [9]. On the contrary, at a more advanced stage of tumor progression loss of IRX2 expression may contribute to tumor cell dissemination and mobilization, as suggested by the previously described association of low IRX2 expression in primary breast tumors with positive DCT status in BM [16] and the here described correlation to positive lymph node status.

The analysis of IRX2 mRNA and protein expression in breast cancer cell lines showed that IRX2 expression is absent in the highly aggressive MDA-MB-231, BT-549 and Hs578T cell lines that are all classified to belong to the basal molecular subtype of breast cancer [24]. These findings are in line with the clinical observation that IRX2 expression is significantly lower in primary tumors belonging to the more aggressive basal subtype. We originally identified low IRX2 to be part of gene signature associated with the presence of DTCs in the bone marrow in patients with luminal breast tumors [16]. However, as low IRX2 is in general associated with the basal subtype of breast cancer, our results indicate that our DTC signature including low IRX2 expression obtained from luminal breast cancer patients defines a more aggressive subpopulation within the luminal group associated with more aggressive traits. Furthermore, as in this study we found a significant correlation between IRX2 and ESR1 expression we therefore assume that IRX2 might function in determining luminal cell differentiation as suggested by other scientists [8].

The biological role of the transcription factor IRX2 in breast epithelial cells is poorly defined and direct transcriptional targets in breast epithelial cells have not been described. We identified several genes whose expression is either positively or inversely correlated with *IRX2* mRNA expression. We found that in particular the expression of several chemokines is inversely correlated with IRX2 expression in breast tumors. To experimentally validate the possible role of IRX2 as a metastasis suppressing protein, we initially aimed to study the potential involvement of the IRX2 transcription factor in breast cancer progression using a RNA interference mediated experimental approach. In our hands neither transfection of small interfering RNAs nor viral transduction of shRNAs yielded a convenient reduction of IRX2 protein expression in breast cancer cell lines, which has already been reported by other researchers [9]. Therefore, we rather stably expressed a c-terminal HA-tagged IRX2 protein in IRX2-deficient BT-549 and Hs578t breast cancer cell lines of basal subtype. Overexpression of the IRX2 transcription factor in BT-549 and Hs578T breast cancer cells leads to concordant repression of *CCL5, CXCL8* and *CXCL10* mRNA expression. CCL5 and CXCL8 were also found to be secreted at reduced levels by BT-549 cells following ectopic IRX2 expression. Elevated levels of CCL5 in plasma and at the primary tumor site of breast cancer patients have been previously associated with progressive and more advanced disease [25] and with axillary lymph node metastasis [26]. Also CXCL8 is overexpressed in invasive breast cancer cells [27] and is believed to play a significant role in the progression of breast cancer [28]. Taken together, these findings suggest that the activity of the IRX2 protein in breast cancer cells might be associated with the control of chemokine expression and that a loss of IRX2 expression in tumor cells might lead to augmented chemokine secretion, which supports mobilization and increases invasiveness of tumor cells. However, it remains unclear if the IRX2 transcription factor is directly involved in the transcriptional regulation of the herein identified chemokines or whether the observed reduced chemokine expression is a secondary effect of IRX2 overexpression. Further studies that also encompasses exact mapping of IRX2 binding sites, are needed to unravel the exact mechanism behind the observed IRX2 mediated down regulation of chemokine expression.

In line with the suggestion that loss of IRX2 expression might contribute to the onset of tumor cell dissemination, we could show that over expressing of IRX2 markedly reduced the motility of breast cancer cells but did not influence cell proliferation. Conditioned media obtained from IRX2 over expressing BT-549 cells is less effective as a chemoattractant than medium from control cells. In addition, when 10 % FCS was used as chemoattractant, we also found that forced IRX2 expression impedes cellular motility independent from the presence of chemokines in conditioned media and most significant results were obtained from the scratch assays. We thus assume that the observed reduction of motility in course of IRX2 overexpression in different assays is based on the generic reduction of chemokines as well as on pleiotropic effects of IRX2 overexpression that still needs to be specified in future studies. Yet, much research remains, including in vivo experiments, to obtain a better understanding of the potential role of the IRX2 transcription factor in the metastatic cascade.

## Conclusion

The obtained results show that low expression of the IRX2 transcription factor occurs mainly in less differentiated, basal breast tumors. We furthermore found that the IRX2 protein inhibits cellular motility of breast cancer cells, supporting the presumptive metastasis suppressing function of the IRX2 protein. In addition, we were able to show that IRX2 over expression represses the secretion of different pro-metastatic chemokines. The loss of IRX2 expression at the primary tumor might therefore contribute to bone metastasis formation by mobilizing cells and rendering them for dissemination.

## Methods

### In silico validation

For in-silico validation the METABRIC gene expression data set consisting of expression results from 1992 breast cancer patients were retrieved from the European Genome-phenome Archive (EGAS00000000083, [29]). Gene expression data on Affymetrix platforms were processed using custom CDF that re-map probes to ENSEMBL transcripts. Using the appropriately pre-processed gene expression values, samples were separated into high-expression and low expression groups at the median and correlated with different clinico-pathological factors provided with gene expression information. A second data set (GEO accession no. GSE6532) [30] consisting of 262 hormone receptor positive and 45 hormone receptor negative breast cancer patients was used to investigate, which genes expression are exhibited correltaed with the IRX2 expression. The dataset was quantile normalized and subsequently genes differentially expressed between the extreme tertials of IRX2 expression were identified using the significance analysis of microarrays (SAM) algorithm with a false discovery rate (FDR) of 5 % and with 1000× repeated permutation [31]. To narrow down the results, in a second, step only transcripts with a fold change > 2 were taken into account.

### Quantitative real-time PCR analysis

qPCR analyses for the patients samples were performed on 150 ng total RNA isolated by RNeasy Micro Kit (Qiagen). For cell line analyses 500 ng total RNA was transcribed using First Strand cDNA Synthesis Kit (Fermentas St. Leon-Rot, Germany) together with 500 ng of universal human reference (UHR, Stratagene, Agilent technologies, Texas USA). Quantitative real-time PCR analyses were performed on Eppendorf Master Cycler using SYBR Green (Fermentas, St. Leon-Rot, Germany) as fluorescence detection method with the following primers; RPLPO-F: TGAGGTCCTCCTTGGTGAACA, RPLPO-R: CCCAGCTCTGGAGAAACTGC, IRX2-F: CCGAGAAACAAAAGCGAAGA, IRX2-R: AGCACGAGTGATCCGTGAG, CCL5-F: CTCGCTGTCATCCTCATTGC, CCL5-R: AAAGCAGCAGGGTGTGGTG, CXCL1-F: CTGAACAGTGACAAATCCAAC, CXCL1-R: CCTAAGCGATGCTCAAACAC, CXCL7-F: GAACTCCGCTGCATGTGTATAA, CXCL7-R: GCAATGGGTTCCTTTCCCGAT, CXCL8-F: GAATTCTCAGCCCTCTTCAAAAAC, CXCL8-R: GCCAAGGAGTGCTAAAGAACTTAG, CXCL10-F: GAAGGGTGAGAAGAGATGTC, CXCL10-R: TAGGGAAGTGATGGGAGAG, CXCL16-F: CCCGCCATCGGTTCAGTTC, CXCL16-R: CCCCGAGTAAGCATGTCCAC. The analyses were performed in triplicates and the mean values were used for each gene. The mRNA levels were normalized to the mRNA level of the ribosomal RPLP0 gene using ΔΔCT-method for quantification. The results, expressed as N-fold differences in target gene expression compared to universal human reference (UHR) or parental cell line expression.

### Cell culture

MDA-MD-231 and SK-BR-3 cells were obtained from ATCC. BT-549, BT-474, T-47D, ZR-75-1 and MDA-MD-468 cells were obtained from Cell Lines Service (Heidelberg). MCF-7 and Hs578T were a friendly gift from Dr. Steven Johnsen (University of Göttingen, Germany). Phoenix amphotropic retroviral packaging cells were a friendly gift from Dr. Volker Assmann (UKE, Hamburg, Germany). CAMA-1 and KPL-1 cells were a friendly gift from Dr K. Iljin (VTT, Espoo, Finland). Cells were grown as monolayers according to standard conditions in either DMEM or RPMI supplemented with 10 % fetal calf serum and 2 mM L-glutamine (Invitrogen) at 37 °C in a humidified atmosphere containing 10 % CO2 or 5 % CO2 respectively. Analysis of cellular viability was done by 3-(4,5-dimethylthiazol-2-yl)-2,5-diphenyltetrazolium bromide (MTT) assay. 5000 cells were plated in quadruplicates and absorbance at 570 nm was measured after 3 h treatment with MTT solution and lysis of cells in isopropanol containing 4 mM HCl and 0.1 % (w/v) NP-40. Lysates were additionally treated with sonicator to achieve complete lysis of cells.

### Expression plasmids and viral transduction

IRX2 coding sequence was amplified (forward-primer: CCGCTGCTCGGCGTGACGCG, reverse-primer: TAGGTAGGGCTGGACGCCC) from cDNA obtained from the breast cancer cell line MCF-7 and cloned into the expression plasmid phCMV3 (Genlantis) using *EcoR*I and *KpN*I restrictions sites. HA-tagged IRX2 cDNA sequence was reamplified and cloned into the retroviral expression plasmid pMXs-IRES-Puro (Cell Biolabs) using *EcoR*I and *Not*I restrictions sites and afterwards the cloned cDNA sequence was verified by sequencing. For production of retroviral particles ψNX-ampho cells were transfected with the retroviral expression plasmid using Lipofectamine 2000 (Invitrogen) according to manufacturer’s suggested protocol. Viral transduction was done with 500 μl viral supernatant added to 50 % confluent recipient cultures in 6-well plates containing 1 ml cell culture medium. Positive selection was achieved 24 h after transduction using puromycin-containing medium (2 μg/ml, Sigma-Aldrich). Cells were subsequently kept under puromycin for 4 days.

### Western blot analysis and antibody production

Whole cell extracts from cultured cells were prepared by direct lysis and sonication of cells in SDS sample buffer containing proteinase and phosphatase inhibitors. Cell extracts were separated on denaturing 8 % polyacrylamide gels and blotted onto PVDF membrane. Detection of IRX2 protein was either done by incubation with an HA specific antibody (Sigma-Aldrich, H6908) or a custom made IRX2 specific antibody. Detection of the HSC70 protein (Santa Cruz, clone B6) was used as loading control. To generate polyclonal antibodies against IRX2, a peptide DDEDDDEEGERGLAPPKPVTSS corresponding to the central region of human IRX2 was synthesized, coupled to keyhole limpet hemocyanin and was then injected into rabbits. IRX2 specific antibodies were isolated by immunoaffinity purification using the corresponding immunizing peptide coupled to a solid support. Reactivity and specificity of the IRX2 specific antibody was verified by Western blot analysis.

### Chemokine antibody array

Conditioned medium from confluent BT-549 and Hs578t cells stably expressing either IRX2 or empty vector were tested for chemokine secretion by chemokine antibody array following the manufacturer’s instructions (R&D Systems Proteome Profiler™ Human Chemokine Array Kit). Estimation of normalized signal intensity was done using ImageJ software.

### In vitro scratch assay

BT-549 cells which were either transduced with IRX2 or empty vector containing retrovirus were plated in a concentration of of 7 × 10^5^ cells and grown to confluence in 6 well plates under standard culture conditions. Afterwards cells were incubated for 24 h in DMEM containing 0.5 % FCS. In vitro scratch assay was performed as described elsewhere [32]. Cell movement was recorded in real time using an Improvision Live Cell Spinning Disk Microscope and data analysis was done using the Volocity® software (Perkin Elmer).

### Transwell migration assay

5 × 10^4^ BT-549 or MDA-MB-231 cells were plated in serum-free DMEM media into the upper chambers of BD Cell Culture Inserts for 24-well plates with 8.0 μm pores (BD Falcon™). In the lower chamber conditioned medium from confluent BT-549 cells stably expressing either IRX2 or empty vector were used as chemoattractant. Plates were incubated at 37 °C, and migration was allowed to proceed for 20 h. Afterwards non migrated cells in the upper chambers were removed with cotton swabs, and the remaining cells were fixed in 4 % paraformaldehyde and stained with crystal violet. Cells were counted by using a Zeiss light microscope. Four fields were counted on each of two filters. Results are expressed as average cells per field.

### Ethical approval

Since all analyses were conducted on previously published expression data from breast cancer patients or on cell lines, no further ethics approval was required for this study.

## Additional files

Additional file 1: Table S1. Genes in primary breast tumors showing exhibited correlation with IRX2 expression and functional annotation of these genes. SAM-analysis (Significance Analysis of Microarrays) on and functional annotation was used on a large published data set of breast cancer patients (GSE6532). (XLS 79 kb) Additional file 2: Figure S1. Gene reporter assay with reporter plasmid containing proximal CCL5 promoter fragment. The reporter plasmid was transfected into BT-549 cells that over express IRX2 and into the control cell line.  For normalization, a co-transfection with the pGL4.74 plasmid containing the Renilla luciferase was performed. Each experiment was performed in triplicate. Error bars represent the standard deviation of the mean. (PDF 18 kb)

## Abbreviations

BM: Bone marrow
CCL5: Chemokine (C-C motif) ligand 5
CCR5: C-C chemokine receptor type 5
CXCL: Chemokine (C-X-C motif) ligand
CpG: CpG dinucleotides
DMEM: Dulbecco’s modified Eagle’s medium
DTC: Disseminated tumor cell
ESR1: Estrogen receptor 1 (alpha)
FCS: Fetal calf serum
FDR: False discovery rate
GEO: Gene expression omnibus
h: Hour
HA: Human influenza hemagglutinin
HER2: Human epidermal growth factor receptor 2
IRX2: Iroquois homeobox 2
mRNA: Messenger RNA
PCR: Polymerase chain reaction
PIK3CA: Phosphatidylinositol-4,5-bisphosphate 3-kinase
qPCR: Quantitative polymerase chain reaction
RPLPO: Ribosomal protein, large, P0
RPMI: Roswell park memorial institute medium
SAM: Significance analysis of microarrays
shRNA: Small hairpin RNA
