# Supplementary material for: Iroquois homeobox 2 suppresses cellular motility and chemokine expression in breast cancer cells
Source: BMC Cancer. 2015 Nov 11;15:896. doi: 10.1186/s12885-015-1907-4 (PMC4642646; doi:10.1186/s12885-015-1907-4)
Supplement: Additional file 2: Figure S1. — Gene reporter assay with reporter plasmid containing proximal CCL5 promoter fragment. The reporter plasmid was transfected into BT-549 cells that over express IRX2 and into the control cell line. For normalization, a co-transfection with the pGL4.74 plasmid containing the Renilla luciferase was performed. Each experiment was performed in triplicate. Error bars represent the standard deviation of the mean. (PDF 18 kb) [file 12885_2015_1907_MOESM2_ESM.pdf]

Supplementary figure S1

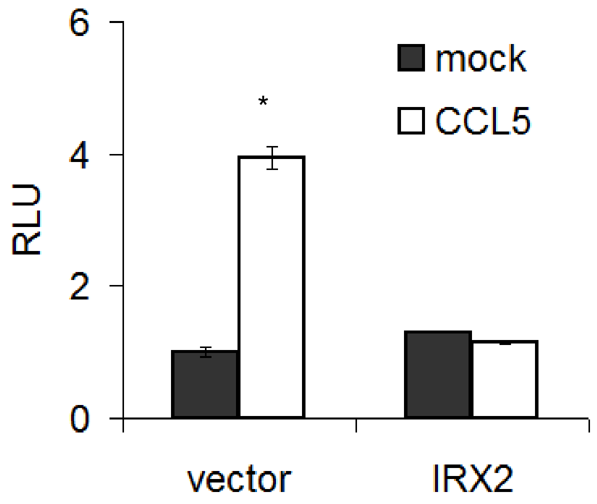

Gene reporter assay with reporter plasmid containing proximal CCL5 promoter fragment. The reporter plasmid was transfected into BT-549 cells that over express IRX2 and into the control cell line. For normalization, a co-transfection with the pGL4.74 plasmid containing the Renilla luciferase was performed. Each experiment was performed in triplicate. Error bars represent the standard deviation of the mean.

The proximal promoter from the human CCL5 gene was PCR-amplified (forward-primer: TCCTGCCTCAACCTCTCGAGTAGC, reverse-primer: TCTTACCTCCTTTCCCTCATCC) and ligated to the firefly luciferase reporter gene by insertion into the XhoI/HindIII cloning sites of the pGL4.10 vector (Promega). BT-549 cells which were either transduced with IRX2 or empty vector containing retrovirus were transiently transfected with the reporter plasmids using Lipofectamine 2000 according to standard protocol. Co-transfection with the pGL4.74 plasmid (Promega) containing the Renilla luciferase cDNA under the control of the HSV-TK promoter served as a control for transfection efficiencies. Forty-eight hours post transfection, cell extracts were assayed in triplicate for luciferase activities using the Dual Luciferase Reporter Assay System (Promega) and a GloMaxTM20/20 luminometer (Promega). Before calculating the fold activation value, luciferase activity of each sample was normalized to the Renilla luciferase activity.
